# Highly efficient microbial inactivation enabled by tunneling charges injected through two-dimensional electronics

**DOI:** 10.1126/sciadv.adl5067

**Published:** 2024-05-03

**Authors:** In-Yong Suh, Zheng-Yang Huo, Jae-Hwan Jung, Donghyeon Kang, Dong-Min Lee, Young-Jun Kim, Bosung Kim, Jinyoung Jeon, Pin Zhao, Jeonghune Shin, SeongMin Kim, Sang-Woo Kim

**Affiliations:** ^1^ School of Advanced Materials Science and Engineering, Sungkyunkwan University (SKKU), Suwon 16419, Republic of Korea.; ^2^ School of Environment and Natural Resources, Institute of Ecological Civilization, Renmin University of China, Beijing 100872, PR China.; ^3^ Thin Film Materials Research Center, Korea Research Institute of Chemical Technology (KRICT), Daejeon 34114, Republic of Korea.; ^4^ Department of Materials Science and Engineering, Center for Human-oriented Triboelectric Energy Harvesting, Yonsei University, Seoul 03722, Republic of Korea.; ^5^ Division of Advanced Materials, Suzhou Institute of Nano-Tech and Nano-Bionics, Chinese Academy of Sciences, Suzhou 215123, PR China.; ^6^ Research and Development Center, SEMS CO., Ltd., Suwon 16229, Republic of Korea.

## Abstract

Airborne pathogens retain prolonged infectious activity once attached to the indoor environment, posing a pervasive threat to public health. Conventional air filters suffer from ineffective inactivation of the physics-separated microorganisms, and the chemical-based antimicrobial materials face challenges of poor stability/efficiency and inefficient viral inactivation. We, therefore, developed a rapid, reliable antimicrobial method against the attached indoor bacteria/viruses using a large-scale tunneling charge–motivated disinfection device fabricated by directly dispersing monolayer graphene on insulators. Free charges can be stably immobilized under the monolayer graphene through the tunneling effect. The stored charges can motivate continuous electron loss of attached microorganisms for accelerated disinfection, overcoming the diffusion limitation of chemical disinfectants. Complete (>99.99%) and broad-spectrum disinfection was achieved <1 min of attachment to the scaled-up device (25 square centimeters), reliably for 72 hours at high temperature (60°C) and humidity (90%). This method can be readily applied to high-touch surfaces in indoor environments for pathogen control.

## Teaser

Tunneling charges under graphene enable consistent electron loss of attached bacteria/viruses for rapid indoor disinfection.

## INTRODUCTION

Respiratory diseases caused by airborne pathogens transmitted through indoor environments are a major public health concern (*1*, *2*). In addition to the recent coronavirus disease 2019 (COVID-19) outbreak, which has infected 10% of the world’s population, airborne pathogens are responsible for tuberculosis, pneumonia, and influenza primarily because of their widespread and highly infectious nature (*3*, *4*). Micro-sized pathogenic aerosols (<1 μm) produced by infected patients during daily activities (e.g., breathing, talking, or coughing) can travel kilometers through the ventilation system and attach to indoor surfaces (e.g., walls, chairs, and floors) (*5*, *6*). These attached pathogens can remain infectious for weeks or longer, posing a significant risk because they can readily infect nearby people in hospitals, high-rise buildings, schools, and public restrooms through accidental inhalation or skin contact (*7*–*9*).

Accordingly, there are two common approaches to preventing indoor airborne infections: (i) blocking the transmission of pathogens using high-efficiency particulate air (HEPA) filters and (ii) applying antimicrobial materials to high-touch surfaces (e.g., elevator buttons and armrests) (*10*–*12*). Compared with physics-separated microbial removal using HEPA filters, which cannot inactivate pathogens and require regular filter replacement, antimicrobial materials hold promise for inactivating attached pathogens to enable early control of pathogenic infections (*13*–*17*). However, commonly used noble metal–based (Ag and Cu) antimicrobial materials suffer from the potential material release under real-world conditions such as high temperatures and humidity during summer, when the incidence of microbial infections is high (*18*). Inorganic antimicrobial materials, such as quaternary ammonium salts and chitosan, can induce bacterial resistance and their feasibility for viral inactivation remains uncertain. In addition, all of these methods that use antimicrobial disinfectants to disrupt bacterial or viral components (e.g., phospholipids, proteins, or DNA) suffer from the limitation of chemical diffusion and the trade-off between disinfection efficiency and reagent dosage (*19*, *20*). Higher doses of disinfectants are required to achieve rapid disinfection, resulting in high manufacturing costs and unwanted leakage of the applied materials (*18*).

The electron transfer–based antimicrobial method allows for the continuous charge transfer of the microorganisms. This process can disrupt the bacterial respiratory system by causing an imbalance of electron flow to eliminate the essential energy generation for cell growth and metabolism (*21*, *22*). Furthermore, interrupted electron transfer in bacteria can trigger the accumulation of reactive oxygen species (ROS), which can damage biocomponents (proteins or DNA) (*23*, *24*). However, the feasibility of the electron transfer antimicrobial method against viruses is still uncertain (*21*). In addition, this method is highly dependent on an additional power supply to provide continuous charge input. This limitation hinders the application potential because the mass deployment of external power supplies, even in the form of batteries, at every high-risk location in certain buildings is impractical. Therefore, the ability to maintain surface charges for extended periods without additional power supply is a potential breakthrough in the practical application of this process.

Electron tunneling allows charges to be injected through a two-dimensional (2D) material and stably stored on an insulator [e.g., silicon dioxide (SiO_2_)] beneath the 2D material for several days without significant dissipation (*25*–*30*). Electron tunneling can be achieved through a contact-based charge injection process in which a bias voltage is applied at the tip of an atomic force microscope (AFM) that scans a graphene monolayer-covered insulator (*26*). However, all of the current charge tunneling devices are designed to operate on microscale transistors (<25 μm^2^), leaving a notable research gap for microbial inactivation in practical applications on larger device areas (at least 10 cm^2^) (*26*, *29*). In addition, the feasibility of inactivating attached bacteria and viruses through such systems remains questionable, as there is limited research on using tunneling charges stored under 2D materials for effective disinfection.

Here, we present the large-scale tunneling charge–motivated disinfection (TCD) device in which graphene monolayers are dispersed on a SiO_2_ insulator for the rapid inactivation of attached bacteria and viruses in indoor environments. Requiring only a one-time charge injection with an applied voltage of 10 V, the tunneling charges are immobilized under the entire dispersed graphene (25 cm^2^; >10^8^ times that of the state-of-the-art device) for 72 hours without significant dissipation, even at high temperatures (60°C) and humidity (90%). On the basis of the antimicrobial mechanism induced by electron transfer, we achieved complete disinfection (99.99% inactivation) of the bacteria and viruses within 1 min of attachment to the device surface.

## RESULTS

### Design of the TCD method

**Fig. 1.**
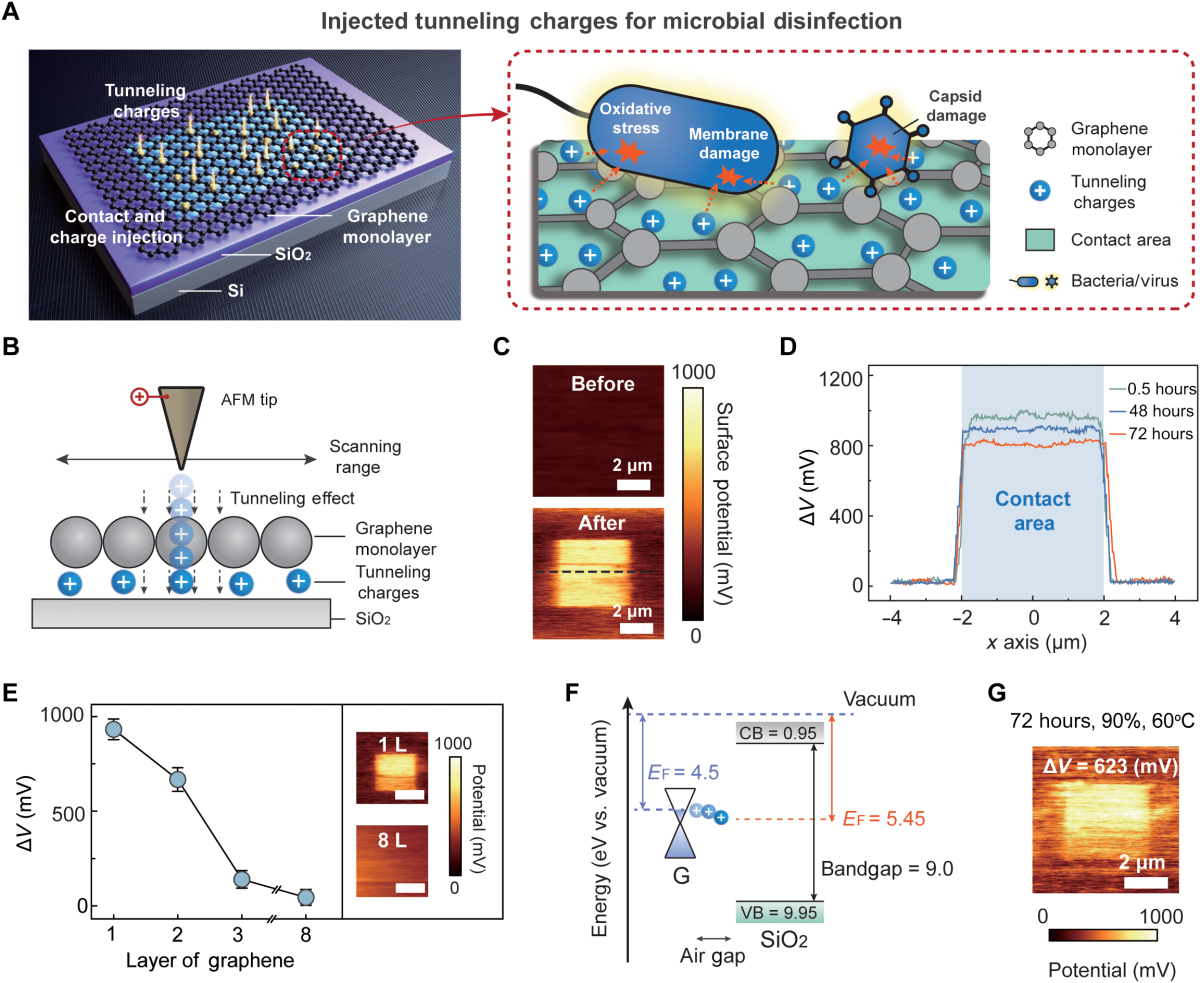
Concept of the TCD method. (**A**) Schematic showing charge injection through the graphene monolayer based on charge tunneling (left) and illustration of microbial inactivation by electron transfer after attachment to the graphene surface (right). (**B**) Electron tunneling occurs when an AFM tip with a bias voltage scans the graphene monolayer. (**C**) Kelvin probe force microscopy (KPFM) image of the charge tunneling device before (top) and after (bottom) scanning by the AFM tip. (**D**) Distribution of tunneling charges measured using KPFM along dashed lines in (C). Δ*V* is the potential difference from charge tunneling. (**E**) Effect of the number of graphene layers (from 1 to 8) on Δ*V*, indicating charge tunneling enabled by the graphene monolayer. The scale bars of the insets in the KPFM measurements are 2 μm. (**F**) Interfacial energy alignment diagrams of graphene and SiO_2_. (**G**) KPFM image of the charge tunneling device in a harsh environment (90% humidity and 60°C temperature) for 72 hours. In (C) to (E), the experiment is performed at a fixed temperature (20°C) and humidity (30%). Error bars represent SD (*n* = 3).

The TCD method incorporates a scaled-up charge tunneling device that enables tunneling charge injection through graphene monolayers when the external electrodes come in contact with them (fig. S1). These tunneling charges can be stored on the surface of the insulator (i.e., SiO_2_) beneath graphene (Fig. 1A, left). Once airborne bacteria and viruses attach to the graphene surface, the stored charges can be readily transferred to the attached microorganisms, damaging the microbial components or disrupting bioactivity (Fig. 1A, right).

There is a notable research gap in which the current charge tunneling devices are primarily targeted toward microscale transistors (<25 μm^2^), whereas microbial inactivation on larger-area devices (at least 10 cm^2^) has received minimal attention. Before constructing a large-scale device for practical applications, we first confirmed the theoretical feasibility of the charge tunneling device for microbial inactivation using an AFM-based microscale transistor (fig. S2) (*26*). This device can be used as a proof of concept for tunneling charge storage, and the stored tunneling charges exhibit strong potential for inactivating the attached microorganisms.

Graphene monolayers were first prepared using chemical vapor deposition (CVD), and then dispersed on a SiO_2_-coated Si wafer (thickness of 300 nm) through wet transfer (fig. S3). Considering the theoretical thickness of the graphene monolayer (0.3 nm) and the measured vertical height of graphene on the SiO_2_ substrate (1.0 nm), an air gap with a thickness of approximately 0.7 nm was formed between graphene and SiO_2_ (fig. S4). As shown in Fig. 1B, a Pt-coated AFM tip with a 10-V bias was applied to scan across the graphene monolayer on the SiO_2_ insulator within an area of 4 μm × 4 μm. Charges from the AFM tip were injected across the graphene monolayer, and then immobilized in the air gap between the graphene and SiO_2_.

The potential of the region scanned by the AFM tip was approximately 1000 mV higher than that of the unscanned region (Fig. 1C). Charges were immobilized in the scanned region for 72 hours without significant dissipation (Fig. 1D). Since free charges can drift and diffuse on a conductive material (i.e., graphene), the reason for charge immobilization is confirmed to be the electron tunneling through the graphene (fig. S5). We further compared the charge immobilization effects of the different graphene layers. The surface potential decreases with an increasing number of graphene layers, indicating that the tunneling charges can only be injected through the monolayer structure (Fig. 1E). In addition, when highly ordered pyrolytic graphite with minimal defects, prepared through exfoliation, was used, a limited number of charges were injected, indicating that the defects in the material facilitated the charge injection (fig. S6).

By fully using the interfacial energy between graphene and SiO_2_, charges can be stably immobilized (*26*). As shown in Fig. 1F, the Fermi level of graphene (4.5 eV) is in the middle of the large bandgap of SiO_2_ (9.0 eV), for which the conduction and valence bands lie at 0.95 and 9.95 eV, respectively. Once the tunneling charges are injected through graphene, they can be stably stored on the SiO_2_ surface because the drifting or diffusion of these charges is difficult. Consequently, the injected tunneling charges can be immobilized for 72 hours (Fig. 1G and fig. S7) even under harsh environmental conditions (temperature of 60°C and humidity of 90%).

### Large-scale charge tunneling for uniform and stable charge retention

**Fig. 2.**
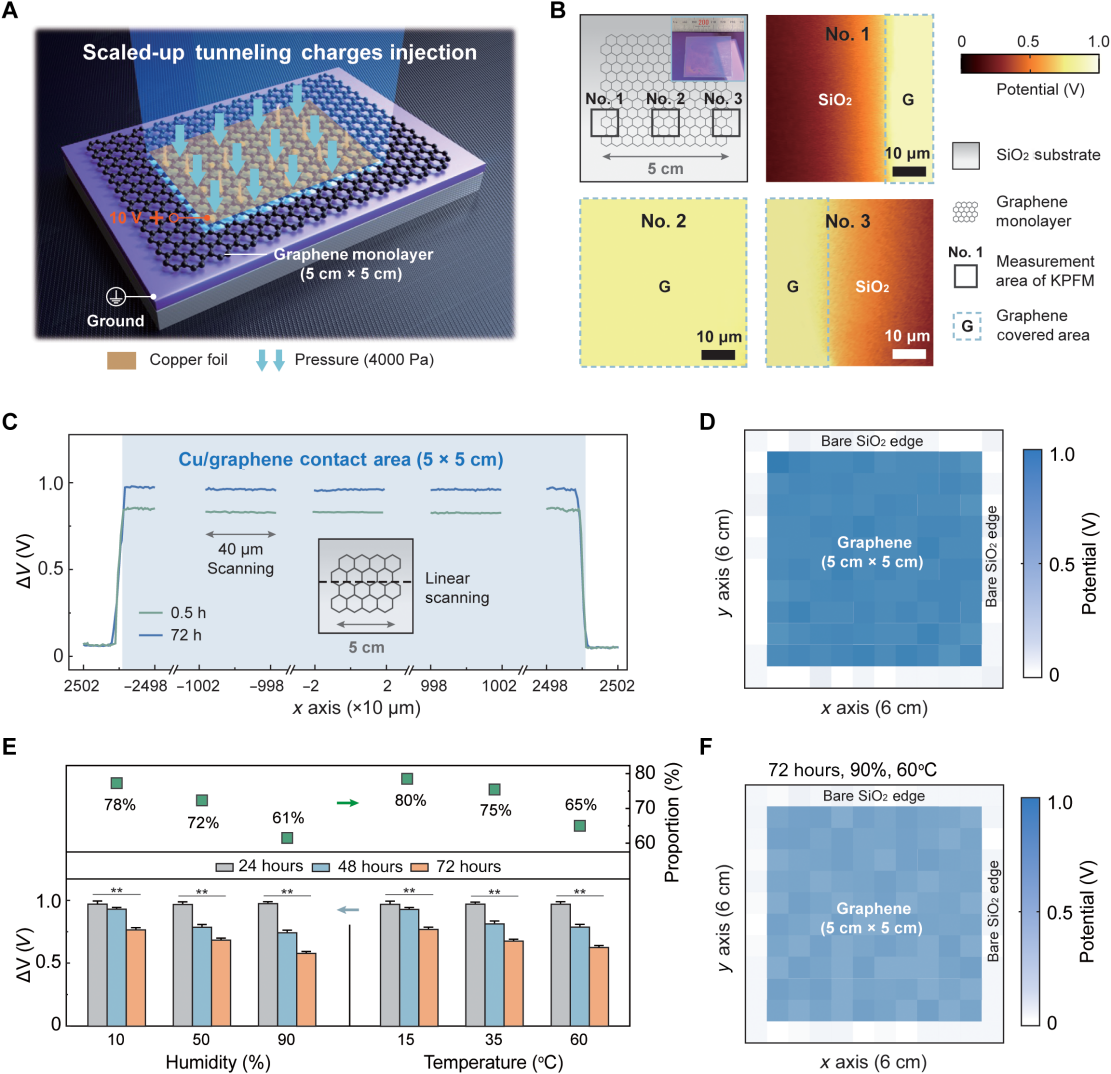
Large-scale charge tunneling for uniform and stable charge retention. (**A**) Schematic illustration of the scaled-up method for a charge tunneling device. The device consists of graphene monolayers covering the SiO_2_ surface (5 cm × 5 cm); charge injection is achieved by applying a bias voltage (10 V) and external pressure (4000 Pa) to a Cu foil. (**B**) KPFM measurements of large-scale charge tunneling devices at different positions (edges and centers). The inset shows the large-scale device. (**C**) Distribution of injected tunneling charges in terms of Δ*V* along the dashed lines in the schematic shown in the inset. (**D**) Distribution of Δ*V* on the device surface divided into 12 × 12 regions (5 mm × 5 mm each) after charging by Cu foil (blue area). (**E**) Effect of humidity (up to 90%) and temperature (up to 60°C) on injected tunneling charges of large-scale devices after 72 hours. (**F**) Distribution of Δ*V* on the device surface divided into 12 × 12 regions after storage in a harsh environment (90% humidity and 60°C temperature) for 72 hours. In (B) to (D), the experiment is performed at a fixed temperature (20°C) and humidity (30%). Error bars represent SD (*n* = 3). Significant differences among groups are indicated by * and ** for *P* < 0.05 and *P* < 0.01, respectively.

Although the proposed charge tunneling device is promising for stable charge retention, its potential application in microbial inactivation is hindered because of the limited scale (16 μm^2^). Therefore, we developed a fit-for-purpose scaled-up charge tunneling device by dispersing graphene monolayers with a controlled area of 5 cm × 5 cm on a SiO_2_-coated Si wafer (fig. S8). Instead of using an AFM tip, which is only feasible for microscale charge injection (several μm^2^), we used a Cu foil (6 cm × 6 cm) with a smooth surface as the conductive electrode and placed it on the fabricated scaled-up device. After applying external voltage (10 V) and pressure (4000 Pa) for 30 s, charges were injected across the graphene monolayer based on the tunneling effect and stored on the surface of the underlying SiO_2_ (Fig. 2A and fig. S9).

The surface potential of the graphene-covered SiO_2_ was approximately 1000 mV higher than that of bare SiO_2_ (Fig. 2, B and C). We further divided the device into 12 × 12 regions (5 mm × 5 mm each) and investigated the distribution of tunneling charges in each region by measuring the surface potential of the central point (40 μm × 40 μm). After 10-V charging, the surface potential was uniform over the entire graphene-coated SiO_2_, whereas the graphene-uncoated bare SiO_2_ showed no significant surface potential (Fig. 2D). Precise charge immobilization without drift indicated successful tunneling charge injection throughout the 5 cm × 5 cm scaled-up device.

Successful charge tunneling also enables stable charge storage, even in harsh environments. More than 60% of the tunneling charges can be stored for 72 hours under the graphene monolayer at a humidity of up to 90% and temperature of up to 60°C (Fig. 2E). The distribution of tunneling charges in each subdivided region (12 × 12) of the device also indicates stable charge storage, with a surface potential of approximately 600 mV uniformly distributed on the graphene-covered SiO_2_ (Fig. 2F). Therefore, the scaled-up charge tunneling device, which requires only a one-time charge injection, successfully enables uniform and stable charge storage throughout the entire graphene-covered region (25 cm^2^). The proposed device exhibits promising potential for the inactivation of attached bacteria or viruses in practical applications.

### Efficacy for microbial inactivation

**Fig. 3.**
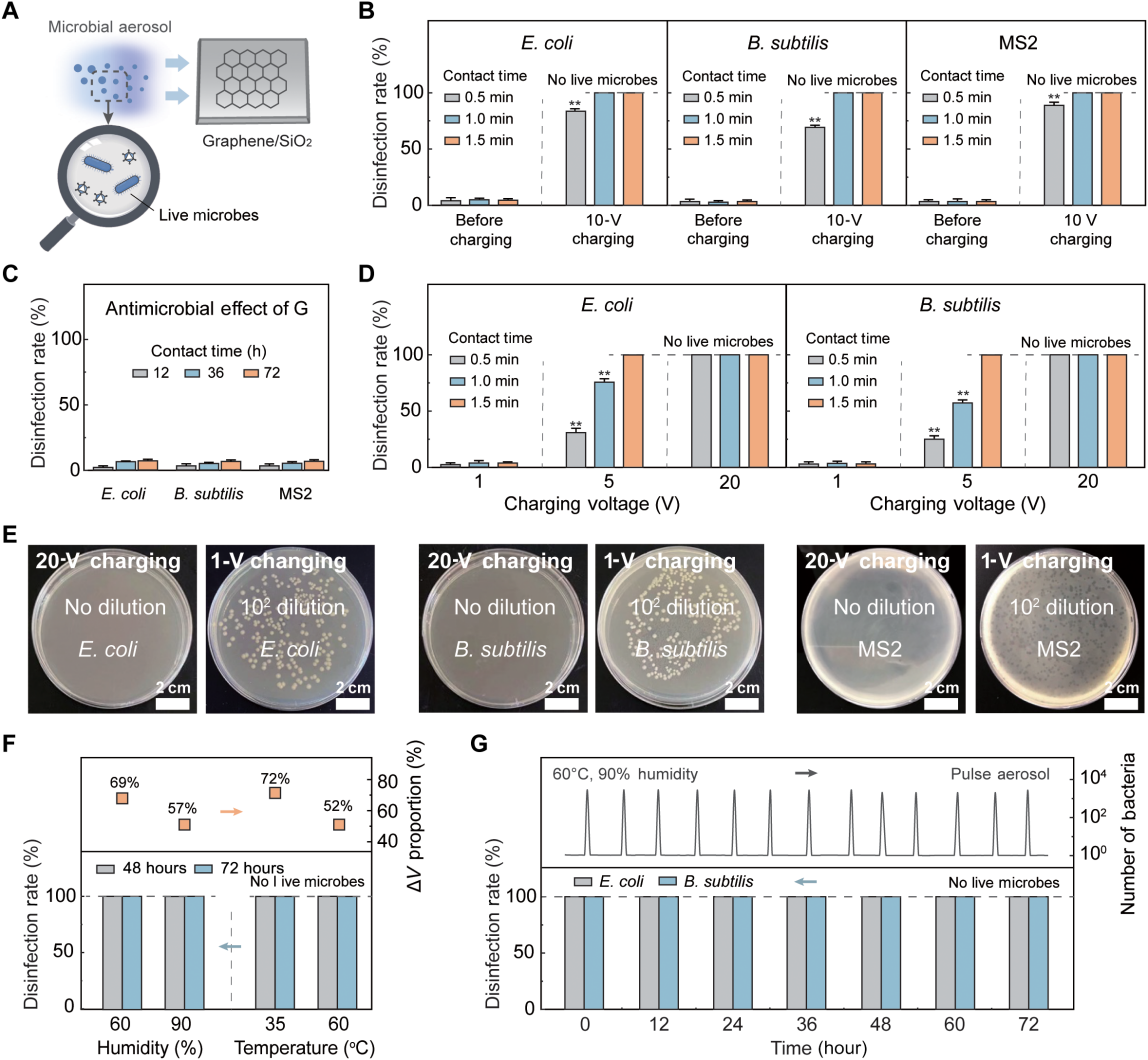
Efficacy of scaled-up TCD device for microbial inactivation. (**A**) Schematic of microbial inactivation process using the scaled-up TCD device, where aerosols containing bacteria or viruses are dispersed on the device surface after charge injection. (**B**) Microbial inactivation efficiency for different model microorganisms [*E. coli* (gram-negative bacterium), *B. subtilis* (gram-positive bacterium), and MS2 bacteriophage (virus)]. (**C**) Effect of graphene on the microbial inactivation efficiency. No charging voltage is applied to the device. (**D**) Effect of charging voltage on microbial inactivation efficiency. (**E**) Images showing the concentration of *E. coli* (left), *B. subtilis* (middle), and MS2 (right) at charging voltages of 20 and 1 V using the scaled-up TCD device. (**F**) Effect of humidity (up to 90%) and temperature (up to 60°C) on the efficacy of the TCD device for *E. coli* inactivation after 48 and 72 hours. (**G**) Efficacy of TCD device for bacterial inactivation when treating intermittently applied aerosols containing *E. coli* or *B. subtilis*. In (B) to (E), the experiment is performed at a fixed temperature (20°C) and humidity (30%). In (E) to (G), the TCD device is charged to 10 V, and microorganisms are measured after 1 min of attachment. Dashed lines indicate that all microorganisms are inactivated (i.e., no live microorganisms detected). Error bars represent SD (*n* = 3). Significant differences among groups are indicated by * and ** for *P* < 0.05 and *P* < 0.01, respectively.

The efficacy of the scaled-up TCD device for microbial inactivation was evaluated using two model bacteria (*Escherichia coli* and *Bacillus subtilis*) representing gram-negative and gram-positive species, respectively, and a model virus (MS2), an *E. coli* bacteriophage as a process surrogate for human enteric viruses (*31*). These microorganisms were dispersed at high concentrations [10^5^ colony-forming units (CFU) ml^−1^ for bacteria or 10^5^ plaque-forming units (PFU) ml^−1^ for viruses] in phosphate-buffered saline (PBS), and then sprayed as aerosols (approximately 0.1 ml) for attachment to the TCD device (Fig. 3A).

After attachment to the scaled-up TCD device for 0.5 min, >70% of the microorganisms were inactivated. The disinfection efficiency of *B. subtilis* (gram-positive bacteria; 72% removal) was lower than that of *E. coli* (gram-negative bacteria, 85% removal) because of the thicker layer of peptidoglycan on the cell wall (*32*). Notably, at longer attachment times, the efficiency of microbial inactivation significantly improved. The TCD device enabled complete disinfection of all tested microorganisms at an attachment time of 1 min, corresponding to >99.99% inactivation (Fig. 3B). We conducted an antimicrobial test without charge injection for an extended period to investigate the potential contribution of graphene’s toxicity to microbial disinfection. After more than 72 hours of attachment to the device without charging, we observed that <10% of the microorganisms were inactivated by contact with graphene, indicating the negligible contribution of graphene to microbial inactivation (Fig. 3C).

The microbial inactivation efficiency improved with the increased tunneling charges that contribute to a high surface potential of graphene. At a charging voltage of 1 V, the surface potential of the TCD device reached 215 mV (fig. S10), which was ineffective for disinfection, with <5% microbial inactivation after 1.5 min of attachment (Fig. 3D and fig. S11). Applying a charging voltage of 5 V resulted in a surface potential of 560 mV, enabling >70% microbial inactivation after 1 min of attachment and complete microbial inactivation (>99.99% microbial inactivation) after 1.5 min of attachment. A higher surface potential of 1130 mV was achieved by increasing the charging voltage to 20 V, which was sufficient for complete microbial inactivation after only 0.5 min of attachment (Fig. 3D and fig. S11). The plating data in Fig. 3E demonstrate a highly efficient microbial inactivation at a charging voltage of 20 V (right) and an ineffective antimicrobial effect at a charging voltage of 1 V (left) after 1.5 min of attachment (Fig. 3E).

The TCD can maintain complete microbial disinfection after finger contact (fig. S12). The adhesion energy between the graphene monolayer and SiO_2_ is 146 mJ m^−2^ based on our simulation result (fig. S13) and previous study (*33*, *34*). In comparison, polydimethylsiloxane, a common material for e-skin and contact lenses with physical properties similar to human skin, shows weak adhesion energy when in contact with the graphene monolayer (7 mJ m^−2^) (*35*). Therefore, graphene monolayers tend to adhere to SiO_2_ and remain stable after human contact. In addition, previous studies have also applied graphene to touch panels and sensors and demonstrated negligible shedding after skin contact, indicating safe application under real conditions (*16*, *17*, *36*, *37*).

The TCD device achieved complete disinfection (99.99% microbial inactivation) and maintained reliability for 72 hours at high temperature (60°C) and humidity (90%) (Fig. 3F and fig. S14). We confirmed the negligible contributions of high temperature (60°C) and humidity (90%) to microbial inactivation after attachment to the uncharged TCD device (fig. S15). Moreover, when microbial bioaerosols were intermittently sprayed onto the TCD device, TCD achieved complete inactivation for all the bacteria and viruses in the bioaerosols after 1 min of attachment and kept reliable for 72 hours (Fig. 3G and fig.S16). Owing to the stable charge retention capability, the TCD device exhibited excellent disinfection efficiency while providing reliable and robust antimicrobial efficacy in practical applications.

### Investigation of antimicrobial mechanisms

**Fig. 4.**
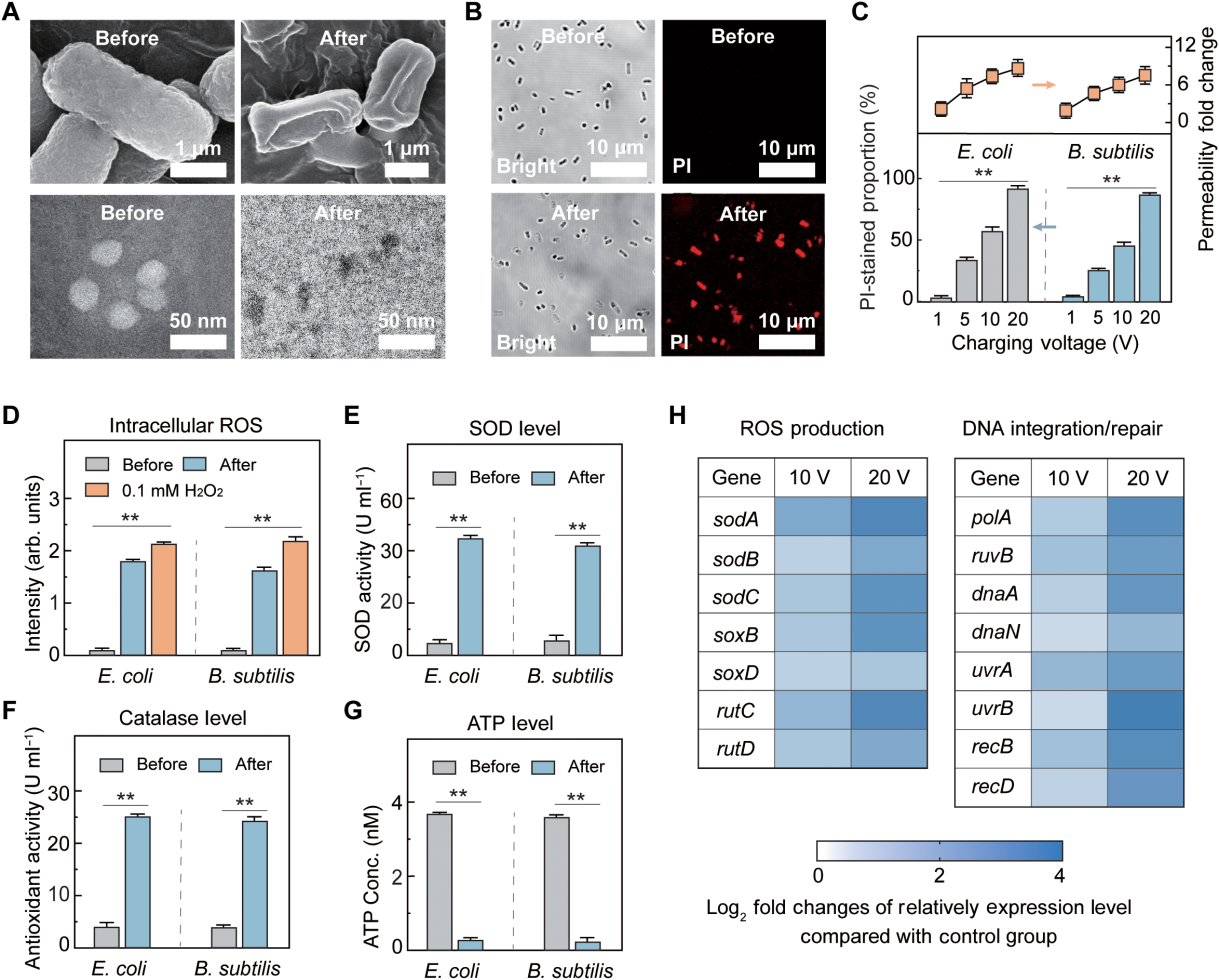
Investigation of antimicrobial mechanism of TCD device. (**A**) SEM images of bacteria (*E. coli*; top) and TEM images of virus (MS2; bottom) before and after attachment to TCD. (**B**) Fluorescence microscopy showing bright-field (total bacteria) and fluorescence (membrane-damaged bacteria) images of *E. coli* before (top) and after (bottom) attachment to TCD. (**C**) Change in membrane permeability of *E. coli* and *B. subtilis* (top) and percentage of bacteria with damaged membrane structures (bottom) after attachment to TCD at different charging voltages (1 to 20 V). (**D**) Intracellular generation of ROS after attachment to TCD. (**E**) Activity of bacterial superoxide dismutase enzyme. (**F**) Activity of bacterial antioxidant enzymes. (**G**) Intracellular adenosine 5′-triphosphate (ATP) levels in TCD-treated bacteria. (**H**) Expression of key genes related to ROS production and DNA integration/repair. Microorganisms were evaluated after 1 min of attachment. In (A), (B), and (D) to (H), the TCD device is charged to 10 V. The experiment is performed at a fixed temperature (20°C) and humidity (30%). Error bars represent SD (*n* = 3). Significant differences among groups are indicated by * and ** for *P* < 0.05 and *P* < 0.01, respectively.

On the basis of the morphological evaluation of the bacteria (scanning electron microscopy; SEM) and viruses (transmission electron microscopy; TEM), it was confirmed that the antimicrobial mechanism of the TCD device was electron transfer. After attachment to the 10 V-charged TCD device for 1 min, the bacteria exhibited significant damage in the form of membrane shrinkage and collapse (Fig. 4A, top). The integrity of the viral capsid was assessed through negative stain-incorporated TEM measurements using phosphotungstic acid, which can penetrate capsid-damaged viruses and show a dark contrast (*31*). After attachment to TCD, viruses (MS2) exhibited damaged capsid, as indicated by the dark contrast in the TEM images (Fig. 4A, bottom).

The compromised bacterial membranes were also evaluated by analyzing membrane permeability using propidium iodide (PI) staining, where PI selectively penetrates bacteria with damaged membrane structures (*32*). Compared to untreated bacteria with intact membrane structures that cannot be stained by PI (Fig. 4B, top), >90% of the attached bacteria showed red fluorescence (Fig. 4B, bottom), indicating a compromised membrane structure after attachment to the TCD. The permeability of the bacterial membrane also increased with the charging voltage. After charging at 1 V, TCD was ineffective for microbial inactivation, with a slight change in bacterial membrane permeability (Fig. 4C). However, charging voltages of 5, 10, and 20 V resulted in two-, six-, and eightfold increases in the membrane permeability of the treated bacteria, respectively, indicating severe membrane damage (Fig. 4C).

Intracellular biochemical reactions were further investigated after bacterial attachment to the TCD device. The amount of intracellular ROS in the TCD-treated bacteria increased significantly to a level comparable to that of the positive control (0.1 mM H_2_O_2_; Fig. 4D and fig. S17). This finding indicates that the respiratory chain is disturbed by the consequent electron loss, leading to the excessive accumulation of oxygen species (*38*). The higher level of bacterial superoxide dismutase (SOD) activity indicated that the TCD-treated bacteria attempted to convert the generated intracellular superoxide into the less oxidative H_2_O_2_ (Fig. 4E and fig. S18). The higher activity of bacterial antioxidant enzymes, which can decompose the generated H_2_O_2_, also confirmed that electron loss triggered bacterial repair response after attachment to TCD (Fig. 4F and fig. S19). The sharp decrease in intracellular adenosine 5′-triphosphate (ATP) levels further indicated the depletion of electron donors, which was attributed to the interruption of the respiratory chain after TCD treatment (Fig. 4G).

Transcriptional analysis revealed that the expression of genes related to ROS production increased, as indicated by SOD (*sodA*, *sodB*, and *sodC*), hydrolases (*rutC* and *rutD*), and sarcosine oxidase (*soxB* and *soxD*). We confirmed that the charge transfer process was ineffective for RNA damage (fig. S20), and the accumulated intracellular ROS after TCD treatment was responsible for the higher gene expression (Fig. 4H and table S1) (*39*). Moreover, after TCD treatment, the bacteria exhibited increased expression of genes related to DNA integration/repair, indicating that the generated ROS caused DNA damage (Fig. 4H and table S2) (*40*–*42*).

**Fig. 5.**
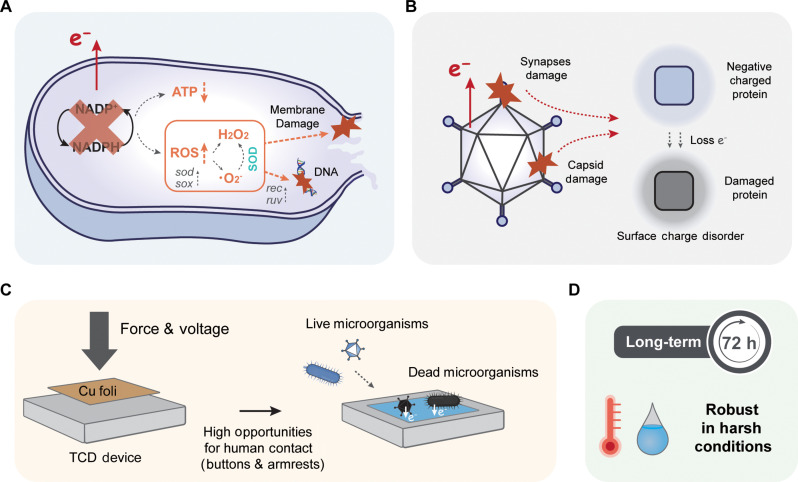
Antimicrobial mechanism of TCD device and its potential application. (**A** and **B**) Schematic illustration of bacterial (A) and viral (B) inactivation mechanisms involved in the TCD process based on electron transfer. (**C**) Potential application scenario of the TCD device on high-touch surfaces in indoor environments (e.g., elevator buttons and armrests in hospitals). (**D**) Unique properties (reliability and robustness) of the TCD device for microbial inactivation in practical applications.

The mechanisms underlying the antimicrobial response of the TCD device are shown in Fig. 5. For the bacterial inactivation (Fig. 5A), the treated cells experience continuous electron loss after their attachment to the TCD device, which interrupts NADPH generation in the respiratory chain owing to the rapid depletion of electron donors. Depletion of electron donors also leads to a sharp decrease in intracellular ATP levels (Fig. 4G) and significant oxidation pressure (Fig. 4, E and H). The generated intracellular ROS further damages the cytoplasm, including the DNA (increased expression of genes related to DNA integration/repair; Fig. 4H) and the membrane (increased permeability; Fig. 4, B and C). Because gram-positive and gram-negative bacteria have similar respiration and ROS generation systems, charge transfer–based disinfection is feasible for different types of bacteria (*21*). For the viruses, failure to infect the hosts (Fig. 4, B and E) and loss of viral capsid integrity (Fig. 4A) occur after attachment to the TCD surface. Therefore, we propose that the antiviral mechanism involves damage to functional viral proteins (i.e., synapses and capsids) via charge transfer (Fig. 5B). Further investigation of the disinfection mechanisms of enveloped viruses, including human immunodeficiency, influenza, and hepatitis B/C viruses, is suggested to fully understand viral inactivation using the TCD method.

TCD device achieves effective, reliable microbial disinfection in terms of high disinfection efficiency, broad-spectrum disinfection of bacteria and viruses, and multiple application scenarios, showing promising potential for addressing the critical need for pathogen control in indoor environments (table S3). The TCD device can be readily applied to high-touch surfaces (e.g., elevator buttons and armrests in hospitals) using one-time charge injection (10 V; Fig. 5C). The feasible disinfection under high temperatures and humidity is critical for pathogen control, in particular during the summer months when the incidence of microbial infections is high (Fig. 5D).

We aim to provide a proof of concept using a graphene monolayer–covered insulator for charge retention that can achieve disinfection. The proposed method can also be adapted to curved surfaces when flexible insulators (polytetrafluoroethylene and polyvinylidene difluoride) serve as substrates for graphene monolayer transfer (*43*, *44*). Furthermore, graphene monolayers can be transferred to SiO_2_ microspheres, which can further cover flexible substrates or textiles and promising for tunneling charge retention (*28*). Other common 2D materials, such as boron nitride (BN) nanosheets, molybdenum disulfide (MoS_2_) nanosheets, and MXenes, are also suitable for charge tunneling when deposited on insulators (*45*–*47*). In addition, the required charging voltage (20 V) can be readily generated from a battery using a commercial boost converter, making it feasible when power supplies are not readily available. Overall, this flexible antimicrobial device offers expanded scenarios for practical application.

## DISCUSSION

In this study, we developed a reliable antimicrobial method to effectively inactivate bacteria and viruses attached to indoor environments using a successfully scaled-up TCD device (size = 25 cm^2^). The stably immobilized tunneling charges under the entire graphene-covered device can be readily injected with an applied voltage of 10 V. Microorganisms attached to a TCD device undergo direct and continuous electron loss, overcoming the diffusion limitations of previous chemical disinfectants and resulting in a highly efficient antimicrobial effect. The TCD device achieved complete bacterial and viral disinfection (>99.99% removal) after 1 min attachment and remained viable for 72 hours at high temperature (60°C) and humidity (90%). This novel TCD antimicrobial device has the potential to address the critical need for reliable bacterial and viral disinfection of indoor environments at a relatively low manufacturing cost of approximately $3.2 for a 25-cm^2^ TCD device (table S4).

## MATERIALS AND METHODS

### Fabrication of TCD device

Graphene monolayers were fabricated on a Cu foil (thickness of 75 μm, Wacopa) through a CVD process (*26*). The temperature of the CVD was increased to 1000°C under an H_2_ atmosphere (10 sccm, 1 torr) for 30 min to clean the surface of the Cu foil. The graphene monolayer was then synthesized under a mixed atmosphere of CH_4_ (20 sccm) and H_2_ (10 sccm) for 30 min (1 torr) at 1000°C. After fabrication, the gas supply was turned off and the chamber was cooled at a targeted cooling rate (160°C min^−1^). Graphene fabricated on the Cu foil was then spin-coated using poly(methyl methacrylate) (PMMA) by controlling the spin rate at 1000 rpm and time at 30 s. After curing at 70°C for 10 min, the PMMA-coated graphene was immersed in a Cu etchant (Transene, type 1) and washed using deionized water. Graphene was transferred onto an insulator (SiO_2_-coated Si wafer) via wet transfer. The graphene-covered area was controlled using masks (for small devices with coverage areas of 16 μm^2^) or tapes (for large devices with coverage areas of 25 cm^2^). The number of deposited graphene layers was controlled by varying the transfer time of the graphene monolayers.

### Charge injection in TCD device and measurement

For the small device, an AFM (XE100, Park) was used to inject the tunneling charges, where the Pt-coated tip scanned the graphene within a targeted region in contact mode with a force of 20 nN and an applied bias voltage of 10 V. For the large device, a 6 cm × 6 cm Cu foil was placed over a 5 cm × 5 cm graphene-covered area on the scaled-up TCD device and a 1-kg weight (corresponding to an external pressure of 4000 Pa) was applied to its upper surface. The positive and negative electrodes of the power supply (Keithley 2400) were connected to a Cu foil and Si wafer, respectively, and a bias voltage in the range of 1 to 20 V was applied for 30 s. After storage for up to 72 hours under controlled temperature (15° to 60°C) and humidity (10 to 90%) conditions, the surface potential of the TCD device was measured using a Kelvin probe force microscope (XE100, Park) containing a Cr/Au-coated silicon tip. For the scaled-up TCD device, an area of 40 μm × 40 μm was scanned at each target position.

### Evaluation of antimicrobial performance

Model bacteria {*E. coli* [American Type Culture Collection (ATCC), 15597] and *B. subtilis* [ATCC, 23857]} were cultured in nutrient broth at 35°C for 8 hours to the log phase, and then rinsed with deionized water by centrifugation (2000*g*). Model viruses [bacteriophage MS2 (ATCC 15597-B1)] were cultured with the host (*E. coli*) at 35°C for 4 hours. High concentrations of bacteria (10^5^ CFU ml^−1^) or viruses (10^5^ PFU ml^−1^) were dispersed in PBS as a feed solution. The microbial feed solution (approximately 0.1 ml) was aerosolized onto the surface of the TCD device. After treatment, the bacteria or viruses were washed from the TCD surface using 10 ml of sterilized deionized water, and the antimicrobial efficiency was evaluated as follows

$$E=\left(N/N_{0}\right)\times 100\%$$

where *N* is the number of live bacteria/viruses after attachment to the TCD device and *N*_0_ is the number of live microbes attached to TCD without charge injection as a control experiment. Live bacteria and viruses were quantified using standard plating or the double agar layer method (*31*).

### Investigation of microbial morphology

The morphology of the *E. coli* before and after attachment to the TCD device was evaluated using SEM (SU8230, Hitachi). After harvesting by centrifugation (2000*g*), the *E. coli* samples were stored in a fixative solution (2% glutaraldehyde) at 4°C for 12 hours, followed by a gradient dehydration process using ethanol. The prepared *E. coli* samples were subjected to a critical point drying process (EM CPD300, Leica). SEM measurements were performed after Au coating (10 nm). The morphology and integrity of MS2 were evaluated using TEM (Tecnai F20, FEI) integrated with phosphotungstic acid staining.

### Evaluation of membrane permeability and intracellular reactions

The permeability of the bacterial membranes was evaluated using the PI staining method and measured using a fluorescence microscope (Axio Imager, Zeiss) and a spectrophotometer (Thermo Fisher Scientific) at 535 nm. Intracellular ROS production was determined using the DCFH-DA ROS assay kit (Beyotime) and measured at 488 nm using a spectrophotometer. H_2_O_2_ (0.1 mM) was used to treat the bacteria as a positive control. The SOD and antioxidant enzyme activities were measured using an SOD colorimetric activity kit (Beyotime) and an antioxidant enzyme assay kit (Beyotime), respectively. Bacterial ATP levels were measured using an ATP assay kit (Beyotime).

### RNA extraction, reverse transcription, and quantitation

Intracellular RNA was extracted using the RNeasy Kit (QIAGEN). The extracted RNA was measured by the spectrophotometer (NanoDrop 2000, Thermo Fisher Scientific) and Bioanalyzer (2100, Agilent) for quality control (fig. S20), followed by reverse transcription to produce cDNA (TransGen Biotech). Functional gene expression (tables S1 and S2) was evaluated using real-time quantitative polymerase chain reaction.

### Statistical analysis

Significant differences were determined using the unpaired Student’s *t* test and are indicated by * and ** for *P* < 0.05 and *P* < 0.01, respectively.

## Supplementary Materials

### This PDF file includes:

Figs. S1 to S20 Tables S1 to S4 References
