# Supplementary material for: Highly efficient microbial inactivation enabled by tunneling charges injected through two-dimensional electronics
Source: Sci Adv. 2024 May 3;10(18):eadl5067. doi: 10.1126/sciadv.adl5067 (PMC11067992; doi:10.1126/sciadv.adl5067)
Supplement: Supplementary file 1 — Figs. S1 to S20 Tables S1 to S4 References [file sciadv.adl5067_sm.pdf]

Science Advances
AAAS

Supplementary Materials for

Highly efficient microbial inactivation enabled by tunneling charges injected through two-dimensional electronics

In-Yong Suh *et al.*

Corresponding author: Zheng-Yang Huo, zhengyanghuo.edu@ruc.edu.cn; Sang-Woo Kim, kimsw1@yonsei.ac.kr

Sci. Adv. **10**, eadl5067 (2024)
DOI: 10.1126/sciadv.adl5067

This PDF file includes:

Figs. S1 to S20
Tables S1 to S4
References

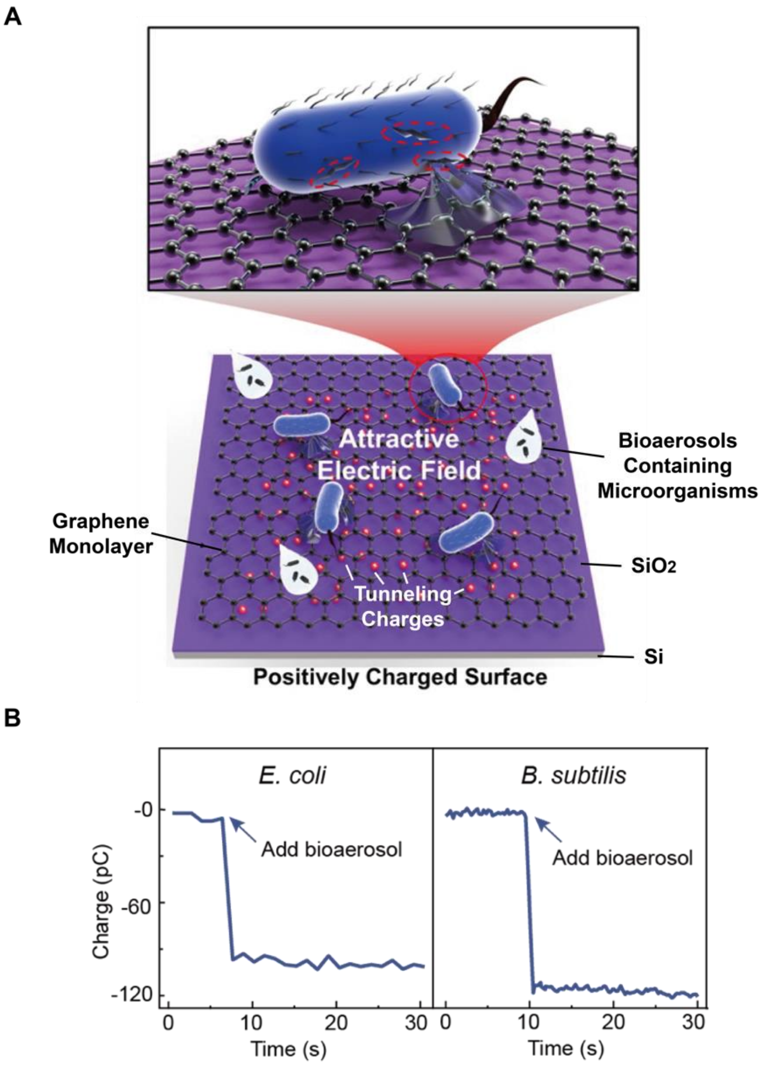

Fig. S1.

Schematics showing the structure of the TCD device. (**A**) The graphene monolayer covers the entire SiO_2 surface charge, and tunneling charges can be injected through the graphene monolayer and stored on the SiO_2 surface. The TCD device enables disinfection once microorganisms are attached. (**B**) Measurement of microbial surface charges in the bioaerosol, indicating that the opposite surface charges between microorganisms (negative) and the surface of the TCD device (positive) can promote the attachment of microorganisms. The potential of the deionized water before and after the collection of the microbial aerosol was measured using a Faraday cup and a multimeter (Keysight, 34470A).

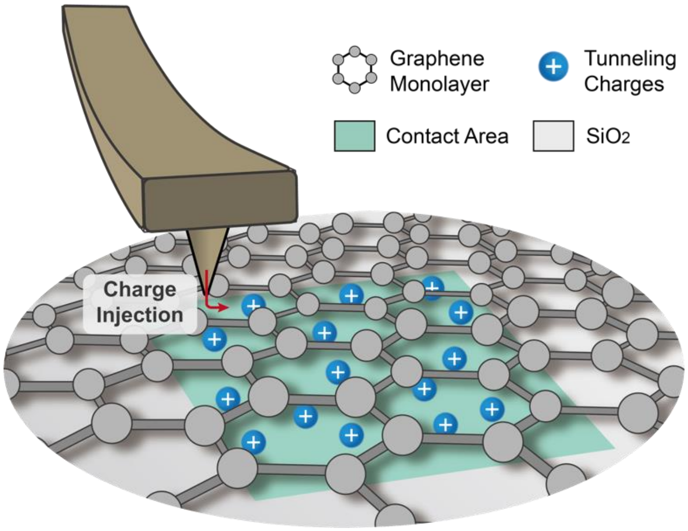

Fig. S2.

Schematics showing the structure of the AFM-based micro-transistor enabling tunneling charge storage. An AFM tip with a 10 V bias was brought into contact with and scanned across the graphene monolayer on the SiO_2 insulator within an area of 4 μm × 4 μm with a force of 20 nN. Charges from the AFM tip were injected across the graphene monolayer, followed by trapping the charges on the SiO_2 surface.

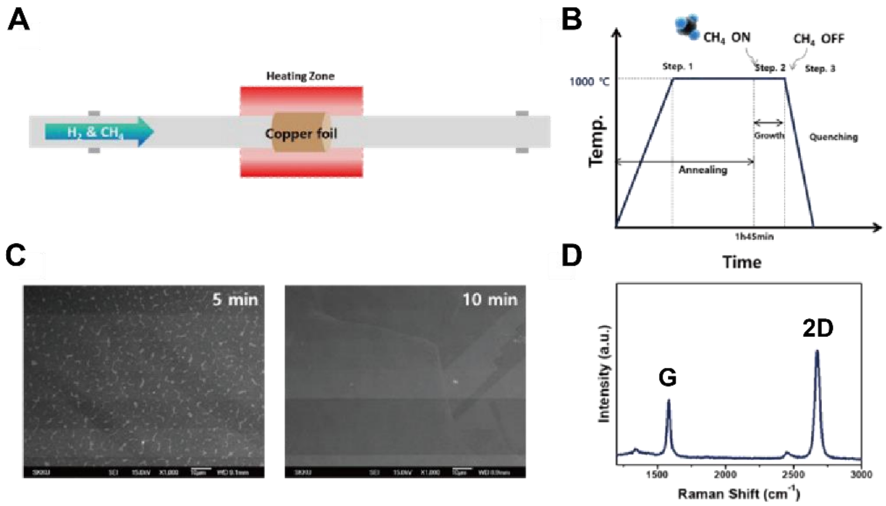

Fig. S3.

Fabrication of graphene monolayers. (**A**) Illustration of the CVD system for the fabrication of graphene monolayers. (**B**) Operating conditions of the CVD process for fabricating graphene monolayers. (**C**) SEM image of the fabricated graphene monolayers on the Cu foil. (**D**) Raman spectra of the graphene monolayer, showing the peak of G and 2D at ~1600 and ~2700 cm^{-1}. The ratios of $\frac{I_G}{I_{2D}} = 0.5$ indicate the as-prepared graphene as a monolayer structure.

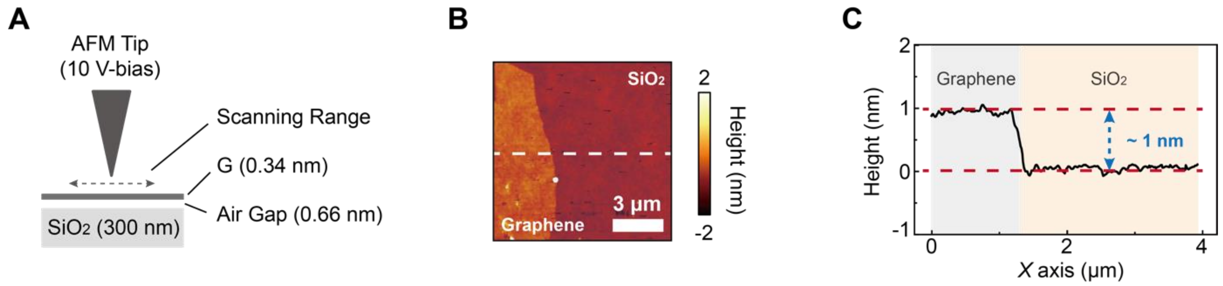

Fig. S4.

Schematic illustration of the air gap thickness of the TCD device. Considering the measured vertical height of graphene on the SiO_2 substrate (1.0 nm) and the theoretical thickness of the graphene monolayer (0.3 nm), the air gap thickness can be estimated to be around 0.7 nm. (**A**) Schematic of the air gap thickness. (**B**) Topography image of as-prepared graphene on SiO_2. (**C**) Linear scan showing the cross-sectional profile along the dashed lines in B.

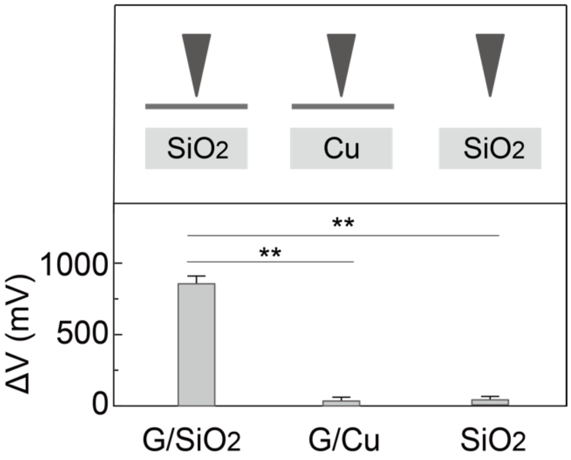

Fig. S5.

Confirmation of charge tunneling. Experiments were performed by comparing the potential difference (ΔV) with and without a graphene monolayer and with a conductive Cu substrate replacing the SiO_2 insulator. Besides the TCD device consisting of graphene monolayers covering the SiO_2, control experiments with graphene monolayers covering a conductive substrate (Cu plate) and bare SiO_2 were set up. Only the TCD device allows charge tunneling and immobilization. Error bars represent the standard deviation (n = 3). Significant differences among groups are indicated by * and ** for $p < 0.05$ and < 0.01, respectively.

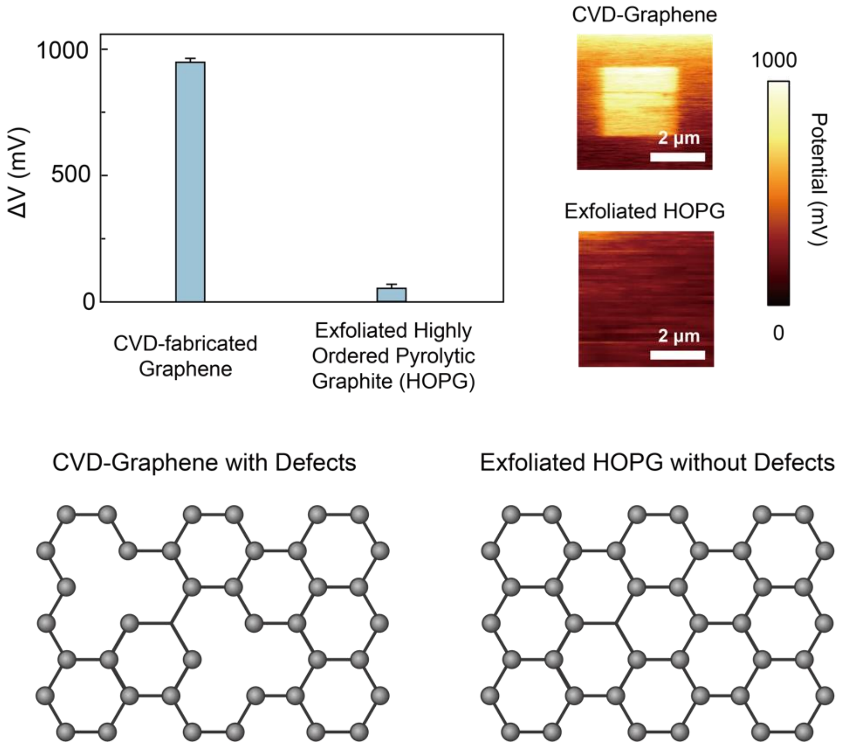

Fig. S6.

Effect of HOPG on charge injection. HOPG was fabricated by the exfoliation process without defects, while the CVD-fabricated graphene had multiple defects. When HOPG is used instead of the CVD-fabricated graphene, few charges can be injected.

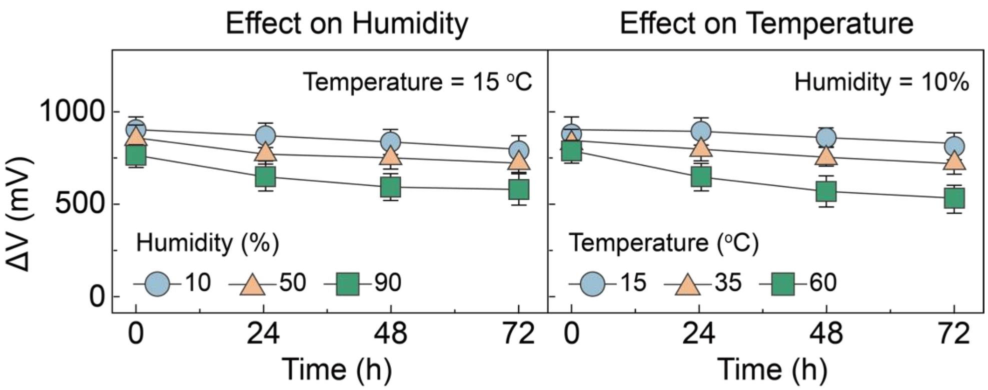

Fig. S7.

Effect of humidity and temperature on charge retention after 72 h. Humidity was controlled from 10 to 90% and temperature was controlled from 15 to 60 °C.

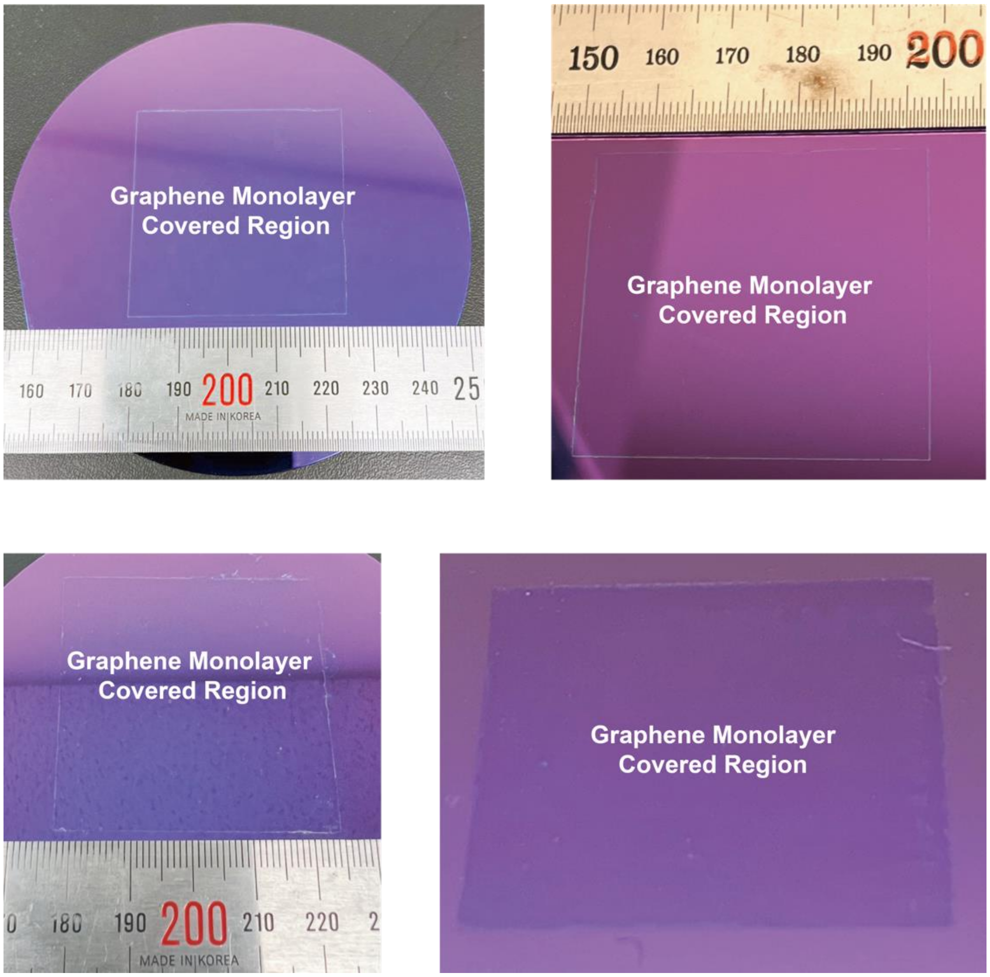

Fig. S8.

Photos of a scaled-up TCD device. The device was fabricated by dispersing graphene monolayers with a controlled area of 5 cm × 5 cm on a SiO_2-coated Si wafer.

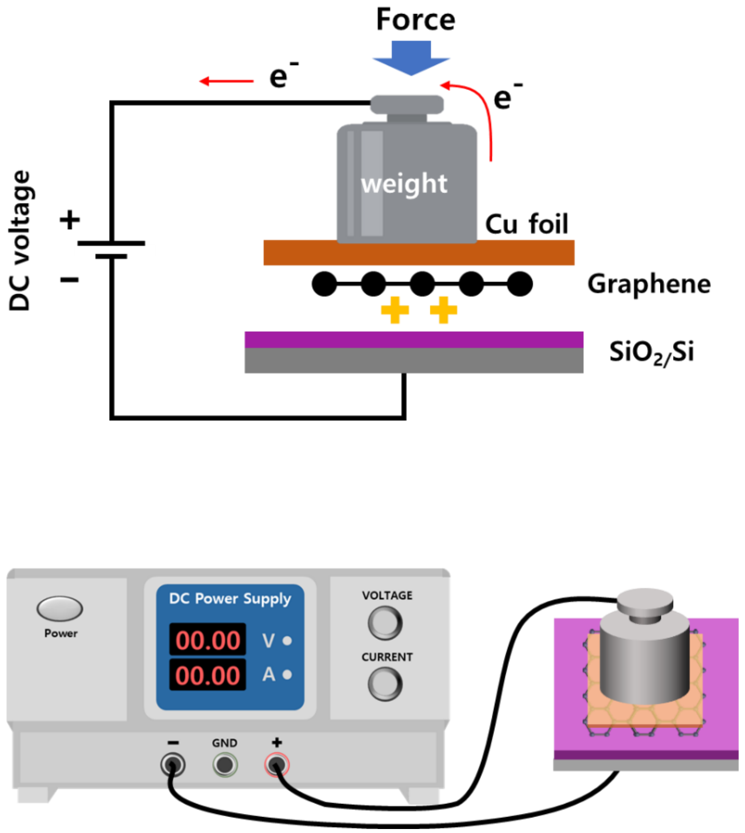

Fig. S9.

Schematic illustration of charge injection for a scaled-up TCD device. The device consisted of a graphene monolayer covering the SiO_2 surface (5 cm × 5 cm), an external power supply, a Cu foil with a bias voltage (10 V), and a 1 kg weight (corresponding to an external pressure of 4000 Pa).

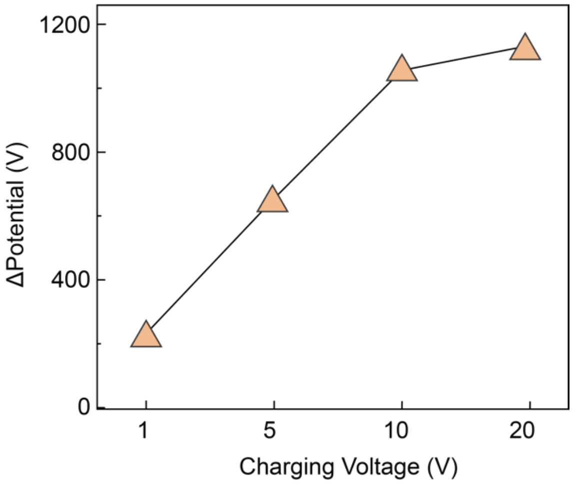

Fig. S10.

Effect of charging voltage (1 − 20 V) on the surface potential of TCD device. The experiment was performed at a fixed temperature (20 °C) and humidity (30%).

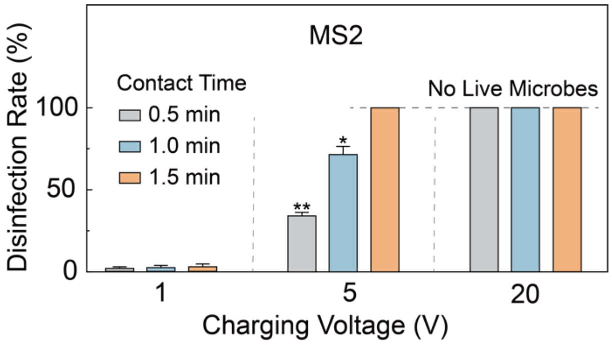

Fig. S11.

Effect of the charging voltage on the viral inactivation efficiency. Dashed lines indicate that all viruses (MS2) tested were inactivated (i.e., no live MS2 was detected). Error bars represent the standard deviation (n = 3). Significant differences among groups are indicated by * and ** for $p <$ 0.05 and < 0.01, respectively.

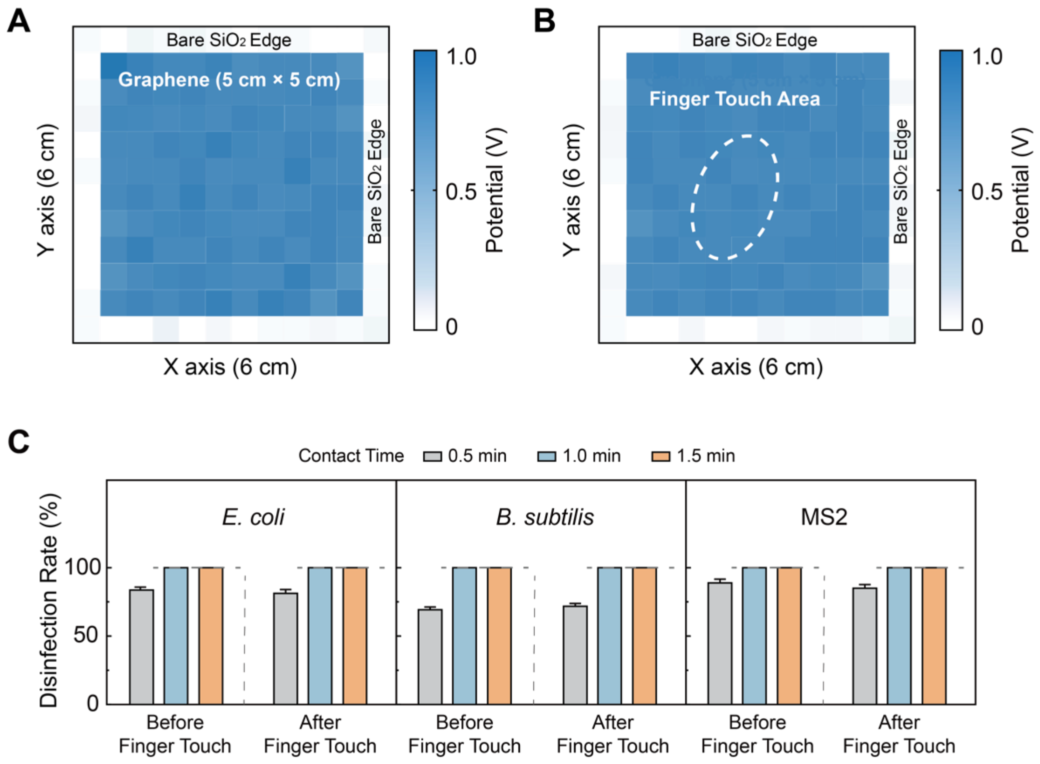

Fig. S12.

Effect of the finger touch on the charge distribution and microbial inactivation efficiency. (**A** and **B**) Distribution of potential (ΔV) from charge tunneling on the device surface before (A) and after (B) finger touch. (**C**) Microbial inactivation efficiency of the device before and after finger touch. Dashed lines indicate that all microorganisms tested were inactivated (i.e., no live microorganisms were detected). Error bars represent the standard deviation (n = 3). Significant differences among groups are indicated by * and ** for $p < 0.05$ and < 0.01, respectively.

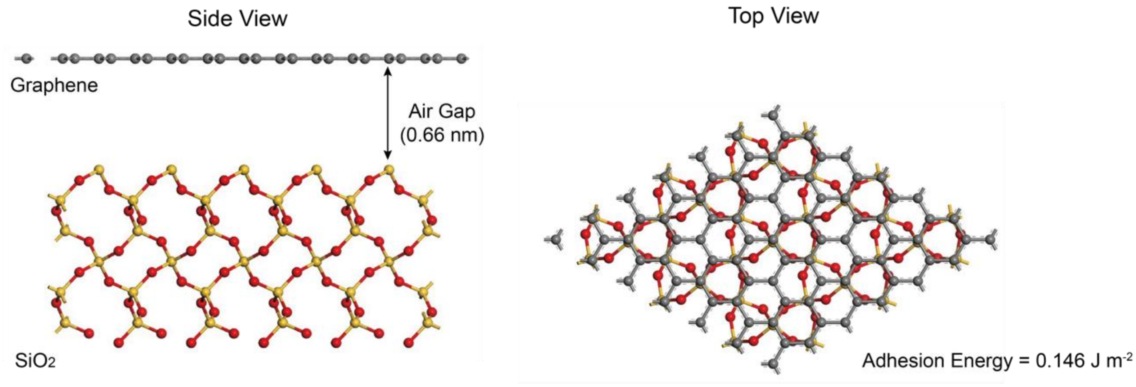

Fig. S13.

Adhesion energy (i.e., binding force) between the graphene monolayer and SiO_2 using DFT simulation. The DFT simulation was performed using Materials Studio (2020, BIOVIA). The Broyden-Fletcher-Goldfarb-Shanno algorithm with the Perdew-Burke-Ernzerhof exchange-correlation function under the generalized gradient approximation was used to optimize the structure. The DFT models consisting of graphene monolayer and SiO_2 were investigated using a plane-wave basis with cutoff energies of 680 and 750 eV, respectively. A standard norm-conserving pseudopotential was used to describe the electron-ion interaction. To avoid spurious interactions between neighboring unit cells in contact and slab models, a vacuum region spanning more than 15 Å was created.

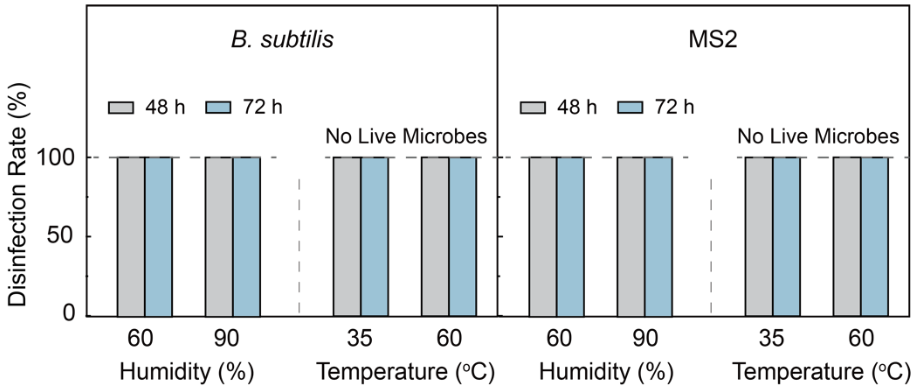

Fig. S14.

Effect of humidity (up to 90%) and temperature (up to 60 °C) on the efficacy of the TCD device for *B. subtilis* and MS2 inactivation after 48 and 72 h. The TCD device was charged at 10 V, and microorganisms were measured after 1 min of attachment. Dashed lines indicate that all microorganisms were inactivated (i.e., no live microorganisms were detected).

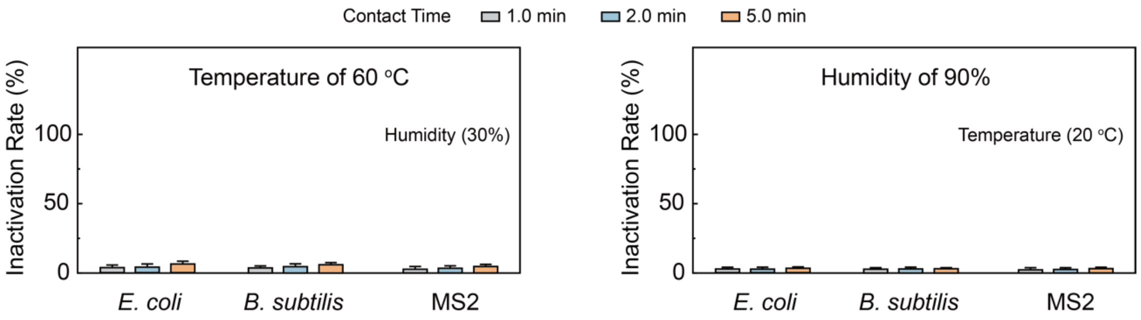

Fig. S15.

Effect of the high temperature (60 °C) or high humidity (90%) on microbial survival. No charging voltage was applied to the TCD device, and microorganisms were measured after attachment for 1.0, 2.0, and 5.0 min. Error bars represent standard deviation (n = 3).

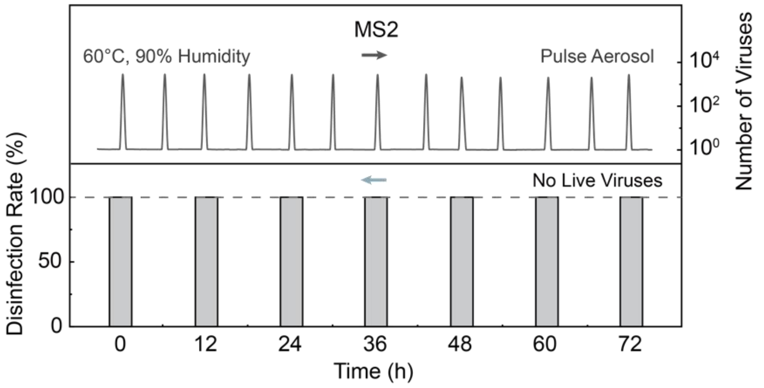

Fig. S16.

Effect of the TCD device for viral inactivation when treating intermittently applied aerosols containing MS2. The experiment was performed at a fixed temperature (20 °C) and humidity (30%). The TCD device was charged at 10 V, and microorganisms were measured after 1 min of attachment. Dashed lines indicate that all viruses (MS2) were inactivated (i.e., no live MS2 was detected).

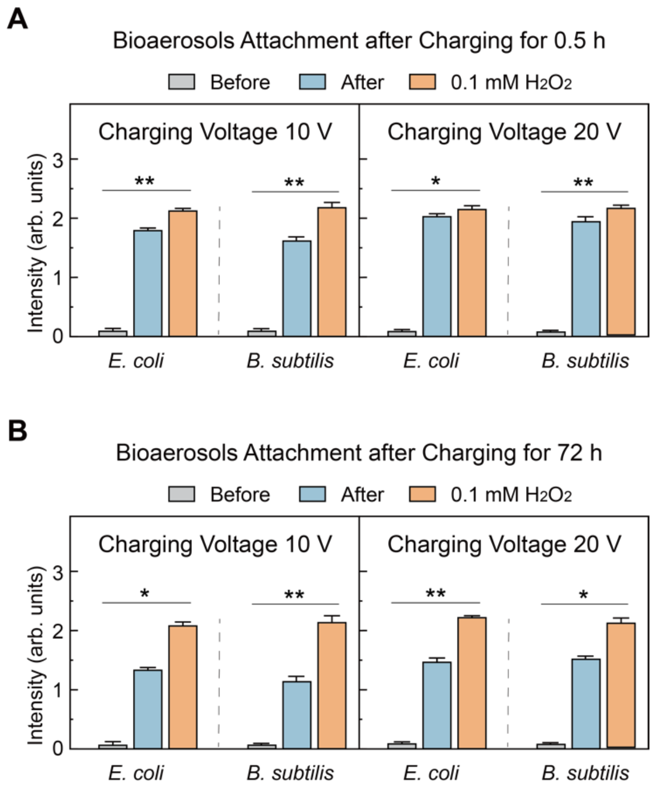

Fig. S17.

Intracellular generation of ROS in bacteria after attachment to the TCD at charging voltages of 10 V and 20 V. (**A**) ROS in bacteria when the TCD was charged for 0.5 h. (**B**) ROS in bacteria when the TCD was charged for 72 h. Microorganisms were evaluated after 1 min of attachment. The experiment was performed at a fixed temperature (20 °C) and humidity (30%). Error bars represent standard deviation (n = 3). Significant differences among groups are indicated by * and ** for $p < 0.05$ and < 0.01, respectively.

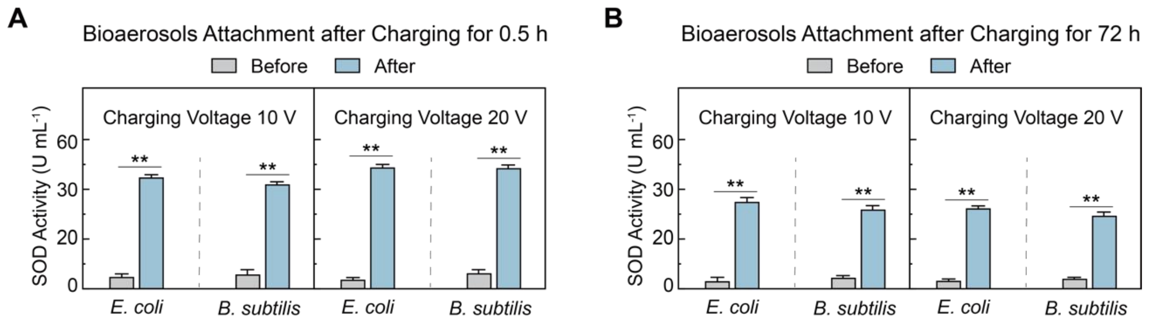

Fig. S18.

The activity of the bacterial SOD enzyme after attachment to the TCD at charging voltages of 10 V and 20 V. (**A**) SOD activity in bacteria when the TCD was charged for 0.5 h. (**B**) SOD activity in bacteria when the TCD was charged for 72 h. Microorganisms were evaluated after 1 min of attachment. The experiment was performed at a fixed temperature (20 °C) and humidity (30%). Error bars represent standard deviation (n = 3). Significant differences among groups are indicated by * and ** for $p < 0.05$ and < 0.01, respectively.

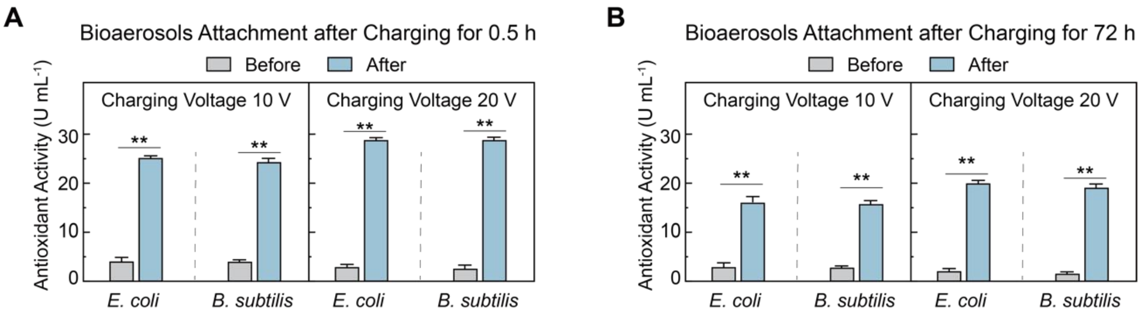

Fig. S19.

The activity of the bacterial antioxidant enzyme after attachment to the TCD at charging voltages of 10 V and 20 V. (**A**) Antioxidant enzyme activity in bacteria when the TCD was charged for 0.5 h. (**B**) Antioxidant enzyme activity in bacteria when the TCD was charged for 72 h. Microorganisms were evaluated after 1 min of attachment. The experiment was performed at a fixed temperature (20 °C) and humidity (30%). Error bars represent standard deviation (n = 3). Significant differences among groups are indicated by * and ** for p < 0.05 and < 0.01, respectively.

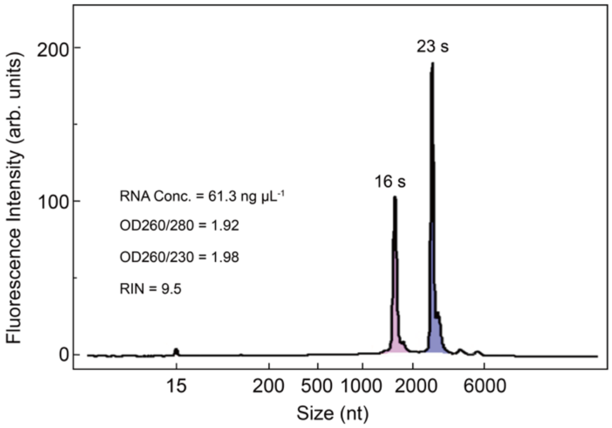

Fig. S20.

Quality control of the extracted RNA. The optical density (OD) of the extracted RNA was measured at 230, 260, and 280 nm. The OD260/280 and OD260/230 indices were higher than 1.9, indicating the high quality of the extracted RNA with complete structure and high purity. The quality of the extracted RNA was also examined using a bioanalyzer. The extracted intracellular RNA showed a high RNA integrity number (RIN) of 9.5 after the attachment of the bacteria to the TCD device. Therefore, the extracted RNA showed high quality in terms of structural integrity, and we confirmed that the charge transfer process was ineffective for RNA damage.

Table S1.

Genes related to ROS production in *E. coli* after the attachment to the TCD device.

Gene Name	Annotation by Clusters of Orthologous Groups	Charging Voltage (10 V)	Charging Voltage (20 V)
sodA	Superoxide Dismutase	1.21	3.42
sodB	Superoxide Dismutase	0.84	2.03
sodC	Superoxide Dismutase	0.93	3.20
soxB	Sarcosine Oxidase Subunit β	0.95	3.25
soxD	Sarcosine Oxidase Subunit β	0.86	1.02
rutC	Pyrimidine Utilization Protein C	1.13	3.38
rutD	Pyrimidine Utilization Protein D	1.05	1.98

Fold change in Fragments Per Kilobase Million (FPKM), comparing with the control group without TCD attachment.

Table S2.

Genes related to DNA integration/repair in *E. coli* after the attachment to the TCD device.

Gene Name	Annotation by Clusters of Orthologous Groups	Charging Voltage (10 V)	Charging Voltage (20 V)
polA	DNA Polymerase I	0.86	3.28
ruvB	Holliday Junction Branch Migration DNA Helicase RuvB	0.96	2.55
dnaA	Chromosomal Replication Initiator Protein DnaA	0.80	3.16
dnaN	DNA Polymerase III Subunit β	0.62	1.69
uvrA	Nucleotide Excision Repair Protein UvrA	0.92	2.93
uvrB	Nucleotide Excision Repair Protein UvrB	0.65	3.67
recB	Exonuclease V Subunit β	0.94	3.26
recD	Exodeoxyribonuclease V Subunit α	0.75	3.18

Fold change in FPKM, compared with the control group without TCD attachment.

Table S3.

Summary of the proposed TCD device and current antimicrobial materials (e.g., textile, film, and rigid devices) for indoor air disinfection.

	Materials	Mechanisms	Feasible for Bacteria	Feasible for Virus	Disinfection Efficiency	Reference
1	Graphene-SiO_2	Charge Transfer	Yes	Yes	1 min, 100% Removal	This work
2	Carbon-TiO_2	Charge Transfer	Yes	No	20 min, 90% Removal	(*21*)
3	Ag-PLGA-PVA Film	Ag Toxicity	Yes	No	3 h, 85% Removal	(*48*)
4	Cu-Cotton Textile	Cu Toxicity	Yes	Yes	3 h, 100% Removal	(*10*)
5	Ag-Cellulose Fiber	Ag Toxicity	Yes	No	24 h, 100% Removal	(*49*)
6	N-halamine-Aerogels	N-halamine Toxicity	Yes	Yes	5 min, 100% Removal	(*50*)
7	TiO_2-Filter	Visible Light Photocatalysis	Yes	No	4 h, >99% Removal	(*51*)
8	MOF-Filter	Visible Light Photocatalysis	Yes	No	2 h, 100% Removal	(*52*)

Table S4.

Cost of material calculation for a TCD device.

Material	Value	Unit price	Cost (USD)
SiO_2-coated Si Wafer	25 cm^2	0.12 USD cm^{-2}	3.00
Cu Foil	25 cm^2	60 USD m^{-2}	0.15
Cost of the main material (25 cm^2)			< 3.20

REFERENCES AND NOTES

1. Y. Liu, Z. Ning, Y. Chen, M. Guo, Y. Liu, N. K. Gali, L. Sun, Y. Duan, J. Cai, D. Westerdahl, X. Liu, K. Xu, K. Ho, H. Kan, Q. Fu, K. Lan, Aerodynamic analysis of SARS-CoV-2 in two Wuhan hospitals. *Nature* **582**, 557–560 (2020).

2. C. C. Wang, K. A. Prather, J. Sznitman, J. L. Jimenez, S. S. Lakdawala, Z. Tufekci, L. C. Marr, Airborne transmission of respiratory viruses. *Science* **373**, eabd9149 (2021).

3. N. H. L. Lenung, Transmissibility and transmission of respiratory viruses. *Nat. Rev. Microbiol.* **19**, 528–545 (2021).

4. G. Bagheri, B. Thiede, B. Hejazi, O. Schlenczek, E. Bodenschatz, An upper bound on one-to-one exposure to infectious human respiratory particles. *Proc. Natl. Acad. Sci. U.S.A.* **118**, e2110117118 (2021).

5. J. R. Port, C. K. Yinda, V. A. Avanzato, J. E. Schulz, M. G. Holbrook, N. Doremalen, C. Shasia, R. J. Fischer, V. J. Munster, Increased small particle aerosol transmission of B.1.1.7 compared with SARS-CoV-2 lineage A in vivo. *Nat. Microbiol.* **7**, 213–223 (2022).

6. T. Merhi, O. Atasi, C. Coetsier, B. Lalanne, K. Roger, Assessing suspension and infectivity times of virus-loaded aerosols involved in airborne transmission. *Proc. Natl. Acad. Sci. U.S.A.* **119**, e2204593119 (2022).

7. S. Moritz, C. Gottschick, J. Horn, M. Popp, S. Langer, B. Klee, O. Purschke, M. Gekle, A. Ihling, F. D. L. Zimmermann, R. Mikolajczyk, The risk of indoor sports and culture events for the transmission of COVID-19. *Nat. Commun.* **12**, 5096 (2021).

8. J. Sznitman, Revisiting airflow and aerosol transport phenomena in the deep lungs with microfluidics. *Chem. Rev.* **122**, 7182–7204 (2021).

9. J. Raymenants, C. Geenen, L. Budts, J. Thibaut, M. Thijssen, H. D. Mulder, S. Gorissen, B. Crasessaerts, L. Laenen, K. Beuselinck, S. Ombelet, E. Keyaerts, E. André, Indoor air surveillance and

factors associated with respiratory pathogen detection in community settings in Belgium. *Nat. Commun.* **14**, 1332 (2023).

10. J. Qian, Q. Dong, K. Chun, D. Zhu, X. Zhang, Y. Mao, J. N. Culver, S. Tai, J. R. German, D. P. Dean, J. T. Miller, L. Wang, T. Wu, T. Li, A. H. Brozena, R. M. Briber, D. K. Milton, W. E. Bentley, L. Hu, Highly stable, antiviral, antibacterial cotton textiles via molecular engineering. *Nat. Nanotechnol.* **18**, 168–176 (2023).

11. M. S. Mauter, I. Zucker, F. Perreault, J. R. Werber, J. H. Kim, M. Elimelech, The role of nanotechnology in tackling global water challenges. *Nat. Sustain.* **1**, 166–175 (2018).

12. Y. Xu, Y. Su, X. Xu, B. Arends, G. Zhao, D. N. Ackerman, H. Huang, S. P. Reid, J. L. Santarpia, C. Kim, Z. Chen, S. Mahmoud, Y. Ling, A. Brown, Q. Chen, G. Huang, J. Xie, Z. Yan, Porous liquid metal-elastomer composites with high leakage resistance and antimicrobial property for skin-interfaced bioelectronics. *Sci. Adv.* **9**, eadf0575 (2023).

13. Y. Guo, C. M. Dundas, X. Zhou, K. P. Johnston, G. Yu, Molecular engineering of hydrogels for rapid water disinfection and sustainable solar vapor generation. *Adv. Mater.* **33**, 2102994 (2021).

14. J. A. Finbloom, P. Raghavan, M. Kwon, B. N. Kharbikar, M. A. Yu, T. A. Desai, Codelivery of synergistic antimicrobials with polyelectrolyte nanocomplexes to treat bacterial biofilms and lung infections. *Sci. Adv.* **9**, eade8039 (2023).

15. Y. Fang, J. Xu, X. Xiao, Y. Zou, X. Zhao, Y. Zhou, J. Chen, A deep-learning-assisted on-mask sensor network for adaptive respiratory monitoring. *Adv. Mater.* **34**, e2200252 (2022).

16. A. Libanori, G. Chen, X. Zhao, Y. Zhou, J. Chen, Smart textiles for personalized healthcare. *Nat. Electron.* **5**, 142–156 (2022).

17. G. Chen, X. Xiao, X. Zhao, T. Tat, M. Bick, J. Chen, Electronic textiles for wearable point-of-care systems. *Chem. Rev.* **122**, 3259–3291 (2022).

18. K. Zheng, M. I. Setyawati, D. T. Leong, J. Xie, Antimicrobial silver nanomaterials. *Coord. Chem. Rev.* **357**, 1–17 (2018).

19. T. Wang, X. Xie, Nanosecond bacteria inactivation realized by locally enhanced electric field treatment. *Nat. Water* **1**, 104–112 (2023).

20. Z. Y. Huo, Y. Du, Z. Chen, Y. H. Wu, H. Y. Hu, Evaluation and prospects of nanomaterial-enabled innovative processes and devices for water disinfection: A state-of-the-art review. *Water Res.* **173**, 115581 (2020).

21. G. Wang, H. Feng, L. Hu, W. Jin, Q. Hao, A. Gao, X. Peng, W. Li, K. Y. Wong, H. Wang, Z. Li, P. K. Chu, An antibacterial platform based on capacitive carbon-doped TiO_2 nanotubes after direct or alternating current charging. *Nat. Commun.* **9**, 2055 (2018).

22. J. Fu, W. Zhu, X. Liu, C. Liang, Y. Zheng, Z. Li, Y. Liang, D. Zheng, S. Zhu, Z. Cui, S. Wu, Self-activating anti-infection implant. *Nat. Commun.* **12**, 6907 (2021).

23. X. Fan, F. Yang, C. Nie, L. Ma, C. Cheng, R. Haag, Biocatalytic nanomaterials: A new pathway for bacterial disinfection. *Adv. Mater.* **33**, e2100637 (2021).

24. J. A. Lemire, J. J. Harrison, R. J. Turner, Antimicrobial activity of metals: Mechanisms, molecular targets and applications. *Nat. Rev. Microbiol.* **11**, 371–384 (2013).

25. Y. Liu, X. Duan, H. J. Shin, S. Park, Y. Huang, X. Duan, Promises and prospects of two-dimensional transistors. *Nature* **591**, 43–53 (2021).

26. S. Kim, T. Y. Kim, K. H. Lee, T. H. Kim, F. A. Cimini, S. K. Kim, R. Hinchet, S. W. Kim, C. Falconi, Rewritable ghost floating gates by tunnelling triboelectrification for two-dimensional electronics. *Nat. Commun.* **8**, 15891 (2017).

27. S. Lin, L. Xu, L. Zhu, X. Chen, Z. L. Wang, Electron transfer in nanoscale contact electrification: Photon excitation effect. *Adv. Mater.* **31**, e1901418 (2019).

28. J. K. Kim, G. H. Han, S. W. Kim, H. J. Kim, R. Purbia, D. M. Lee, J. K. Kim, H. J. Hwang, H. C. Song, D. Choi, S. W. Kim, Z. L. Wang, J. M. Baik, Electric-field-driven interfacial trapping of drifting triboelectric charges via contact electrification. *Energy Environ. Sci.* **16**, 598–609 (2023).

29. Z. Bai, Y. Xiao, Q. Luo, M. Li, G. Peng, Z. Zhu, F. Luo, M. Zhu, S. Qin, K. Novoselov, Highly tunable carrier tunneling in vertical graphene-WS_2-graphene van der Waals heterostructures. *ACS Nano* **16**, 7880–7889 (2022).

30. K. Leng, L. Wang, Y. Shao, I. Abdelwahab, G. Grinblat, I. Verzhbitskiy, R. Li, Y. Cai, X. Chi, W. Fu, P. Song, A. Rusydi, G. Eda, S. A. Maier, K. P. Loh, Electron tunneling at the molecularly thin 2D perovskite and graphene van der Waals interface. *Nat. Commun.* **11**, 5483 (2020).

31. Z.-Y. Huo, Y.-J. Kim, I.-Y. Suh, D.-M. Lee, J. H. Lee, Y. Du, S. Wang, H.-J. Yoon, S. W. Kim, Triboelectrification induced self-powered microbial disinfection using nanowire-enhanced localized electric field. *Nat. Commun.* **12**, 3693 (2021).

32. Z.-Y. Huo, L. R. Winter, X.-X. Wang, Y. Du, Y.-H. Wu, U. Hübner, H. Y. Hu, M. Elimelech, Synergistic nanowire-enhanced electroporation and electrochlorination for highly efficient water disinfection. *Environ. Sci. Technol.* **56**, 10925–10934 (2022).

33. S. P. Koenig, N. G. Boddeti, M. L. Dunn, J. S. Bunch, Bunch, Ultrastrong adhesion of graphene membranes. *Nat. Nanotechnol.* **6**, 543–546 (2011).

34. W. Gao, P. Xiao, G. Henkelman, K. M. Liechti, R. Huang, Interfacial adhesion between graphene and silicon dioxide by density functional theory with van der Waals corrections. *J. Phys. D Appl. Phys.* **47**, 255301 (2014).

35. S. Scharfenberg, D. Z. Rocklin, C. Chialvo, R. L. Weaver, P. M. Goldbart, N. Mason, Probing the mechanical properties of graphene using a corrugated elastic substrate. *Appl. Phys. Lett.* **98**, 091908 (2011).

36. D.-M. Lee, M. Kang, I. Hyun, B. J. Park, H. J. Kim, S. H. Nam, H. J. Yoon, H. Ryu, H. M Park, B. O. Choi, S.-W. Kim, An on-demand bioresorbable neurostimulator. *Nat. Commun.* **14**, 7315 (2023).

37. X. Meng, X. Xiao, S. Jeon, D. Kim, B.-J. Park, Y.-J. Kim, N. Rubab, S. Kim, S.-W. Kim, An ultrasound-driven bioadhesive triboelectric nanogenerator for instant wound sealing and electrically accelerated healing in emergencies. *Adv. Mater.* **35**, e2209054 (2023).

38. Z. Y. Huo, Y. Yang, J. M. Jeong, X. Wang, H. Zhang, M. Wei, K. Dai, P. Xiong, S. W. Kim, Self-powered disinfection using triboelectric, conductive wires of metal-organic frameworks. *Nano Lett.* **23**, 3090–3097 (2023).

39. S. Zhang, Y. Wang, J. Lu, Z. Yu, H. Song, P. L. Bond, J. Guo, Chlorine disinfection facilitates natural transformation through ROS-mediated oxidative stress. *ISME J.* **15**, 2969–2985 (2021).

40. R. Branicky, Y. Wang, A. Khaki, J. L. Liu, M. Kramer-Drauberg, S. Hekimi, Stimulation of RAS-dependent ROS signaling extends longevity by modulating a developmental program of global gene expression. *Sci. Adv.* **8**, eadc9851 (2022).

41. S. Bai, J. Wang, K. Yang, C. Zhou, Y. Xu, J. Song, Y. Gu, Z. Chen, M. Wang, C. Shoen, B. Andrade, M. Cynamon, K. Zhou, H. Wang, Q. Cai, E. Oldfield, S. C. Zimmerman, Y. Bai, X. Feng, A polymeric approach toward resistance-resistant antimicrobial agent with dual-selective mechanisms of action. *Sci. Adv.* **7**, eabc9917 (2021).

42. P. Chen, J. Lang, Y. Zhou, A. Khlyustova, Z. Zhang, X. Ma, S. Liu, Y. Cheng, R. Yang, An imidazolium-based zwitterionic polymer for antiviral and antibacterial dual functional coatings. *Sci. Adv.* **8**, eabl8812 (2022).

43. L. G. P. Martinsa, Y. Song, T. Zeng, M. S. Dresselhaus, J. Kong, P. T. Araujo, Direct transfer of graphene onto flexible substrates. *Proc. Natl. Acad. Sci. U.S.A.* **110**, 17762–17767 (2013).

44. V. P. Pham, H. S. Jang, D. Whang, J. Y. Choi, Direct growth of graphene on rigid and flexible substrates: Progress, applications, and challenges. *Chem. Soc. Rev.* **46**, 6276–6300 (2017).

45. L. Britnell, R. V. Gorbachev, R. Jalil, B. D. Belle, F. Schedin, M. I. Katsnelson, L. Eaves, S. V. Morozov, A. S. Mayorov, N. M. R. Peres, A. H. C. Neto, J. Leist, A. K. Geim, L. A. Ponomarenko, K. S. Novoselov, Electron tunneling through ultrathin boron nitride crystalline barriers. *Nano Lett.* **12**, 1707–1710 (2012).

46. H. Wang, C. C. Huang, T. Polcar, Controllable tunneling triboelectrification of two-dimensional chemical vapor deposited MoS_2. *Sci. Rep.* **9**, 334 (2019).

47. S. Guha, A. Kabiraj, S. Mahapatra, High-throughput design of functional-engineered MXene transistors with low-resistive contacts. *NPJ Comput. Mater.* **8**, 202 (2022).

48. X. Peng, K. Dong, C. Ye, Y. Jiang, S. Zhai, R. Cheng, D. Liu, X. Gao, J. Wang, Z. L. Wang, A breathable, biodegradable, antibacterial, and self-powered electronic skin based on all-nanofiber triboelectric nanogenerators. *Sci. Adv.* **6**, eaba9624 (2020).

49. X. He, H. Zou, Z. Geng, X. Wang, W. Ding, F. Hu, Y. Zi, C. Xu, S. L. Zhang, H. Yu, M. Xu, W. Zhang, C. Lu, Z. L. Wang, A hierarchically nanostructured cellulose fiber-based triboelectric nanogenerator for self-powered healthcare products. *Adv. Funct. Mater.* **28**, 1805540 (2018).

50. F. Wang, Y. Si, J. Yu, B. Ding, Tailoring nanonets-engineered superflexible nanofibrous aerogels with hierarchical cage-like architecture enables renewable antimicrobial air filtration. *Adv. Funct. Mater.* **31**, 2107223 (2021).

51. K. J. Heo, S. B. Jeong, J. Shin, G. B. Hwang, H. S. Ko, Y. Kim, D. Y. Choi, J. H. Jung, Water-repellent TiO_2-organic dye-based air filters for efficient visible-light-activated photochemical inactivation against bioaerosols. *Nano Lett.* **21**, 1576–1583 (2021).

52. P. Li, J. Li, X. Feng, J. Li, Y. Hao, J. Zhang, H. Wang, A. Yin, J. Zhou, X. Ma, B. Wang, Metal-organic frameworks with photocatalytic bactericidal activity for integrated air cleaning. *Nat. Commun.* **10**, 2177 (2019).
